# Machine Learning on Human Muscle Transcriptomic Data for Biomarker Discovery and Tissue-Specific Drug Target Identification

**DOI:** 10.3389/fgene.2018.00242

**Published:** 2018-07-12

**Authors:** Polina Mamoshina, Marina Volosnikova, Ivan V. Ozerov, Eugene Lane, Ekaterina Skibina, Franco Cortese, Alex Zhavoronkov

**Affiliations:** ^1^Pharmaceutical Artificial Intelligence Department, Insilico Medicine, Inc., Baltimore, MD, United States; ^2^Department of Computer Science, University of Oxford, Oxford, United Kingdom; ^3^Computer Technologies Lab, Saint Petersburg State University of Information Technologies, Mechanics and Optics, Saint Petersburg, Russia; ^4^Biogerontology Research Foundation, London, United Kingdom; ^5^Buck Institute for Research on Aging, Novato, CA, United States

**Keywords:** aging, biomarkers of aging, deep learning, machine learning, pathway analysis, target identification

## Abstract

For the past several decades, research in understanding the molecular basis of human muscle aging has progressed significantly. However, the development of accessible tissue-specific biomarkers of human muscle aging that may be measured to evaluate the effectiveness of therapeutic interventions is still a major challenge. Here we present a method for tracking age-related changes of human skeletal muscle. We analyzed publicly available gene expression profiles of young and old tissue from healthy donors. Differential gene expression and pathway analysis were performed to compare signatures of young and old muscle tissue and to preprocess the resulting data for a set of machine learning algorithms. Our study confirms the established mechanisms of human skeletal muscle aging, including dysregulation of cytosolic Ca^2+^ homeostasis, PPAR signaling and neurotransmitter recycling along with IGFR and PI3K-Akt-mTOR signaling. Applying several supervised machine learning techniques, including neural networks, we built a panel of tissue-specific biomarkers of aging. Our predictive model achieved 0.91 Pearson correlation with respect to the actual age values of the muscle tissue samples, and a mean absolute error of 6.19 years on the test set. The performance of models was also evaluated on gene expression samples of the skeletal muscles from the Gene expression Genotype-Tissue Expression (GTEx) project. The best model achieved the accuracy of 0.80 with respect to the actual age bin prediction on the external validation set. Furthermore, we demonstrated that aging biomarkers can be used to identify new molecular targets for tissue-specific anti-aging therapies.

## Introduction

As the world population is experiencing an unprecedented increase in the percentage of people over 65 years of age, the impact of age-related pathologies such as sarcopenia become greater. Sarcopenia significantly impacts quality of life and is one of the hallmarks of aging. The growing body of evidence and experimental data on life extension of model organisms suggests the feasibility of finding interventions promoting human longevity (Moskalev et al., 2015), and understanding the molecular mechanisms of sarcopenia could help in designing desirable interventions. However, the restricted experimental possibilities of studying human aging coupled with the overall low translation rate from model organisms to the human clinic in other therapeutic areas (Mak et al., 2014) complicates the search for desirable anti-aging therapies, with only a few geroprotectors (i.e., anti-aging molecules) having shown potential efficacy in humans to date (Aliper et al., 2016, 2017; Thomas and Gregg, 2017). Biomarkers of aging, or aging clocks, are promising tools empowering human aging research with the ability to track aging changes and evaluate possible rejuvenating treatments (Horvath, 2013; Peters et al., 2015; Putin et al., 2016; Mamoshina et al., 2018), without resorting to long and costly longitudinal clinical studies evaluating the effects of geroprotective interventions upon long-term incidence of age-related morbidity, or lifespan itself. As such, biomarkers of aging have the potential to substantially increase the feasibility of clinically evaluating possible geroprotective interventions.

To date, data-driven approaches have been utilized in a variety biomedical applications (Mamoshina et al., 2016), including drug discovery (Kadurin et al., 2017a,b), and biomarker development (Putin et al., 2016; Mamoshina et al., 2018), both of which provide an attractive alternative to more conventional types of data analysis as they do not require prior knowledge of biological dependencies. With this in mind, we have combined machine learning with a parametric signaling pathway analysis tool in order to identify and categorize the signaling pathway changes in aged skeletal muscles and to propose a muscle-tissue specific panel of aging biomarkers, along with a novel target identification tool for muscle anti-aging therapies.

We first applied a state of the art signaling pathway analysis algorithm, iPANDA, to compare transcriptomic signatures of “old” and “young” muscles. Then, we applied several machine learning methods widely used in bioinformatics including elastic net regression, support vector machines, random forest and neural networks to predict the age of samples based on their transcriptomic signatures. By incorporating feature importance analysis, we used trained age predictors to identify key genes associated with muscle aging. We propose elevation of cytosolic Ca^2+^, PPAR signaling and neurotransmitter recycling as the key signaling axes that contribute to the muscle aging process along with IGFR pathway activation accompanied by PI3K-Akt-mTOR signaling axis activation.

## Materials and methods

### Data

Gene expression profiles were collected from the publicly available repositories Gene Expression Omnibus (https://www.ncbi.nlm.nih.gov/geo/) and ArrayExpress (https://www.ebi.ac.uk/arrayexpress/). In total, we analyzed 545 transcriptomic samples, labeled according to the chronological age of the tissue samples' donors, from 12 datasets GSE1428 (Giresi et al., 2005), GSE25941 (Raue et al., 2012), GSE28392 (Raue et al., 2012), GSE28422 (Raue et al., 2012), GSE38718 (Liu et al., 2013), GSE40645 (Gheorghe et al., 2014), GSE47881 (Phillips et al., 2013), GSE47969 (Sood, 2015), GSE59880 (Timmons et al., 2010; Keller et al., 2011; Sood, 2015), GSE80 (Welle et al., 2002) (Table S1).

As external validation data, we downloaded gene expression profiles of skeletal muscles from the Genotype-Tissue Expression (GTEx) project portal (www.gtexportal.org). Samples (*n* = 564) were mapped to the age bins and sex of donors.

### Cross-platform normalization

We used the *distran* function with the number of assay clusters to use set to 6 and “kmeans” clustering algorithm from the R CONOR package (https://github.com/jcrudy/CONOR) for the cross-platform normalization of gene expression data of the GTEx data. Because most of samples belong to the 50–59 and 60–69 age bins, we performed it by age groups to avoid bias.

### Supervised machine learning models

#### Train and test set design

Models were trained on expression values of 7,682 common genes (Table S2). The dataset was split into training and testing sets at an 80/20 ratio, and were normalized with “normalize.quantiles” from the “preprocessCore” package (Bolstad et al., 2003).

#### Regression model implementation

We adapted five machine learning methods for the age prediction task: ElasticNet, Support Vector Machines, k-Nearest Neighbors, Random Forests and feed-forward neural networks (Deep Feature Selection model, Li et al., 2016). For all shallow models we used their implementation in scikit-learn. To build and train deep models (i.e., networks with more than 3 layers) we used the Keras python library with tensorflow backend. All age predicting models were optimized using a grid search of the hyperparameter space. We trained the models with five-fold cross validation to compensate for overfitting and to receive more robust performance metrics. All optimized model parameters are supplied in Table 1.

**Table 1:** The performance of age predicting models trained on expression profiles on the test set.

**Model**	**Best parameters**	***r [f; m]***	***R* ^2^**	***MAE (years)***	***ε-accuracy***
k-nearest neighbors	Auto algorithm; N of neighbors of 5; distance as weights	0.78 [0.79; 0.76]	0.64 [0.67; 0.62]	9.73 [9.5; 9.8]	0.58 [0.60; 0.56]
Random forest	N trees of 700 with max depth of 50	0.84 [0.88; 0.82]	0.69 [0.71; 0.66]	9.54 [9.2; 9.7]	0.66 [0.67; 0.63]
ElasticNet	Alpha of 0.001 and L1 ratio of 0.2	0.88 [0.92; 0.87]	0.78 [0.84; 0.76]	7.37 [7.0; 7.66]	0.83 [0.84; 0.79]
Support vector machines	Linear kernel with cost of 0.01	0.91 [0.95; 0.80]	0.83 [0.89; 0.80]	7.20 [6.1; 6.5]	0.87 [0.89; 0.85]
Deep feature selection model	Adam optimizer with lr of 10^−5^; 3 hidden layers (512, 256, 128 units); l1, l2 and frobenius norm regularizers; ELU activation function; Dropout of 0.5	0.91 [0.96; 0.89]	0.83 [0.92; 0.78]	6.24 [5.6; 8.1]	0.80 [0.83, 0. 78]

*r for Pearson correlation coefficient; R^2^ for coefficient of determination; MAE for mean absolute error, that shows the average disagreement between actual chronological and predicted ages; ε-accuracy the accuracy of prediction within a period, which was calculated for ε of 10 years; f for metrics calculated only for female samples and m for male*.

#### Model evaluation

The following metrics were used to evaluate the accuracy of age prediction models:

1. Pearson correlation coefficient:
  $$\begin{array}{l} r=\frac{\sum_{i=1}^{N}\;(x_{i}-\bar{x})(y_{i}-\bar{y})}{\sqrt{\sum_{i=1}^{N}\;{\left(x_{i}-\bar{x}\right)}^{2}}\sqrt{\sum_{i=1}^{N}\;{\left(y_{i}-\bar{y}\right)}^{2}}}.\\ \; \end{array}$$
  where *x*_*i*_ is chronological age value x and is the mean of *x*, y_i_ is predicted age value and y is the mean of *y*, N is number of samples. r shows the strength of a linear association between predicted and actual age.
2. Coefficient of determination: $R^{2}=1-\frac{\overset{N}{\underset{i=1}{\sum }}{\left({ŷ}_{i}-y_{i}\right)}^{2}}{\overset{N}{\underset{i=1}{\sum }}{\left(y_{i}-\bar{y}\right)}^{2}},$ where *y*_*i*_ is the real value, ŷ_*i*_ is the predicted value, and $\underline{\bar{y}}$ is the mean of *y*. *R*^2^ shows the percentage of variance explained by the regression between predicted and actual age.
3. Mean absolute error: $MAE=\;\frac{1}{N}\overset{N}{\underset{i=1}{\sum }}|{ŷ}_{i}-y_{i}|;\;$where ŷ_*i*_ is a predicted age, *y*_*i*_ is an age value, and *N* is a number of samples. *MAE* demonstrates average disagreement between the chronological age and the predicted age.
4. $\varepsilon -accuracy=\frac{\overset{N}{\underset{i=1}{\sum }}1_{A}\left({ŷ}_{i}\right)}{N}$, where *A* = [*y*_*i*_−ε; *y*_*i*_+ε], ŷ_*i*_ is an age prediction of the model, and *y*_*i*_ is a true age value. For instance, if epsilon (ε) is 5 and the DNN model predicts an age of 55 but the real age is 50 or 60, then according to epsilon accuracy, such a sample would be considered correctly classified.

We used *multiclass.roc* function from the pROC R package to calculate multiclass area under the receiver operating characteristic curve for the accuracy (mAUC) of age bin prediction.

#### Feature importance analysis

In the present study, we explore several methods to evaluate the importance of features (genes) on age prediction. We first ranked genes by absolute values of their regression coefficients for an ElasticNet model. We then applied the Random Forest feature importance algorithm to extract the Gini importance value of each gene. Next, we explored the relative importance values assigned to genes by the deep feature selection model, averaging the importance values of genes for the five-fold cross validation process.

In addition to feature importance ranking, we also explored the wrapper method, which we have successfully applied previously in the context of identifying the most important blood markers for age prediction (Putin et al., 2016; Mamoshina et al., 2018). We applied the same technique in the present study, with some modification. Here we explored random permutations of vectors of gene expression values along with increased (by log_2_ fold changes of 3) and decreased (log_2_ fold changes of −3) gene expression values.

In case of random permutations, ${x^{\prime }}_{i}=rand\;\left(x\right)$, where *x* is a vector of expression of *i* gene.

In case of a direct increase or decrease, ${x^{\prime }}_{i}=x\times 2^{f}$, where *x* is a vector of expression of *i* gene and *f* is a fold change of 3 and −3 respectively.

Therefore feature importance value for the gene *i* is calculated as $FI_{i}=\frac{\overset{k}{\underset{m=1}{\sum }}\frac{R^{2}\left(Y,\hat{Y}\right)}{R^{2}\left(Y,\;{\hat{Y}}^{\prime }\right)}}{k}$, where $\hat{Y}$ is a vector of predicted value of age and ${\hat{Y}}^{\prime }$ is a vector predicted values of age after permutations, *k* is a number of cross-validation folds and, in this case, equals to 5.

We used Support Vector Machine algorithm as an age predicting model. Each model predicts age after a modification of gene expression values and assigns an importance coefficient to the gene based on the accuracy of age prediction. Afterwards, scores obtained on the validation sets are summed, and each gene-associated importance factor is averaged to yield a final value.

Borda count algorithm was applied to summarize all six ranks derived from age predicting models, and the rank of genes sorted by absolute log_2_ fold change values derived from differential expression analysis, in order to obtain the final importance rank of genes.

### Signaling pathway analysis

Raw gene expression data were normalized with RMA method (Bolstad et al., 2003). Nine independent datasets from the NCBI GEO database, including GSE80, GSE1428, GSE28392, GSE47881, GSE47969, GSE59880, GSE28422, GSE38718, and GSE25941 were carefully selected for the analysis. For each dataset the groups corresponding to the samples from the “old” and the “young” individuals, respectively, were constructed. The samples from individuals 16–30 years old were considered “young,” while individuals over 60 years old were considered “old.” In all the following parts of the analysis the “old” group was used as a reference and the young group was compared to it. In order to obtain the list of differentially expressed genes, data were processed using the R “limma” package (Ritchie et al., 2015). Benjamini-Hochberg FDR adjustment was applied to the *p*-values (Benjamini and Hochberg, 1995). The pathway level analysis was performed using the iPANDA software suite (Ozerov et al., 2016). Positive and negative iPANDA scores indicated up- and down-regulation of the pathway, respectively. The pathway database used for the analysis included 1,856 annotated and manually curated signaling pathway maps from KEGG, Reactome and NCI-PID and SA Biosciences (http://saweb2.sabiosciences.com/pathwaycentral.php) collections (Kanehisa and Goto, 2000; Schaefer et al., 2009; Croft et al., 2014).

## Results

**Figure 1:**
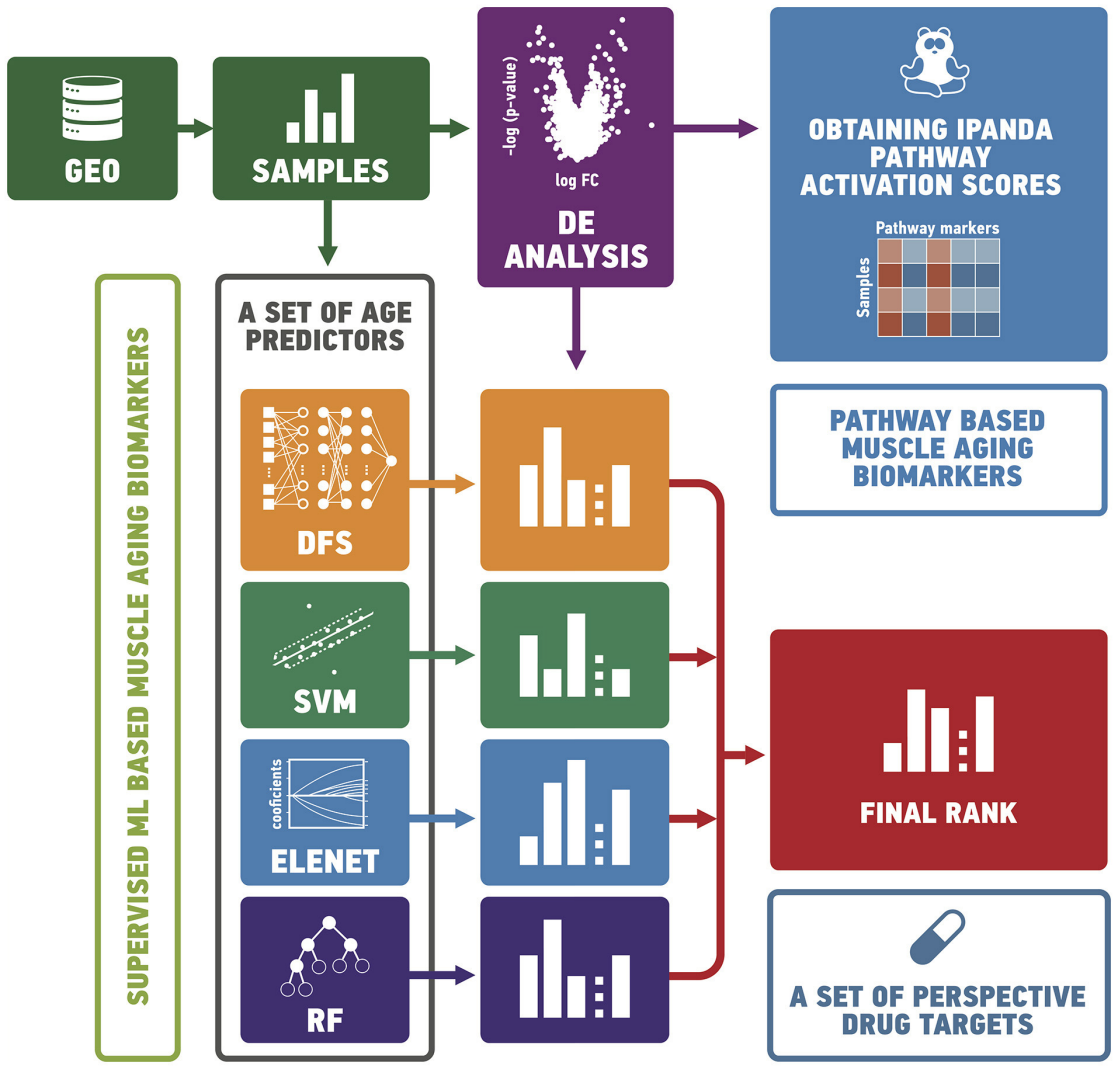
In order to study the effects of aging in human skeletal muscle, we collected gene expression profiles of 19–89 year old individuals from publicly-available datasets. We split samples into “old” and “young” groups and analyzed them using differential gene expression and pathway analysis. We then trained a set of supervised models to predict the age of samples. Finally, we ranked genes according to their importance for age prediction using Borda count over rank values obtained by ElasticNet, Random Forest, Deep Feature Selection and wrapper algorithms. GEO, gene expression omnibus; DE, differential expression analysis; DFS, deep feature selection model; SVM, support vector machines; ELNET, ElasticNet; RF, random forest.

In order to study the effects of aging in human skeletal muscle, we obtained 545 gene expression profiles of 19–89 age individuals from publicly-available datasets. We first split samples into “old” and “young” groups and analyzed them using differential gene expression analysis and pathway analysis (see Figure 1). We then trained a set of supervised models to predict the age of samples. Finally, we ranked genes according to their importance for age prediction using Borda count over rank values obtained by ElasticNet, Random Forest, Deep Feature Selection and wrapper algorithms.

### Gene expression and signaling pathway analysis

To profile the signalome differences between young and old skeletal muscle, we applied the iPANDA algorithm (Ozerov et al., 2016) to normalized gene expression data. An analysis of 9 muscle datasets obtained from the publicly available NCBI GEO database has revealed various age-related effects.

**Figure 2:**
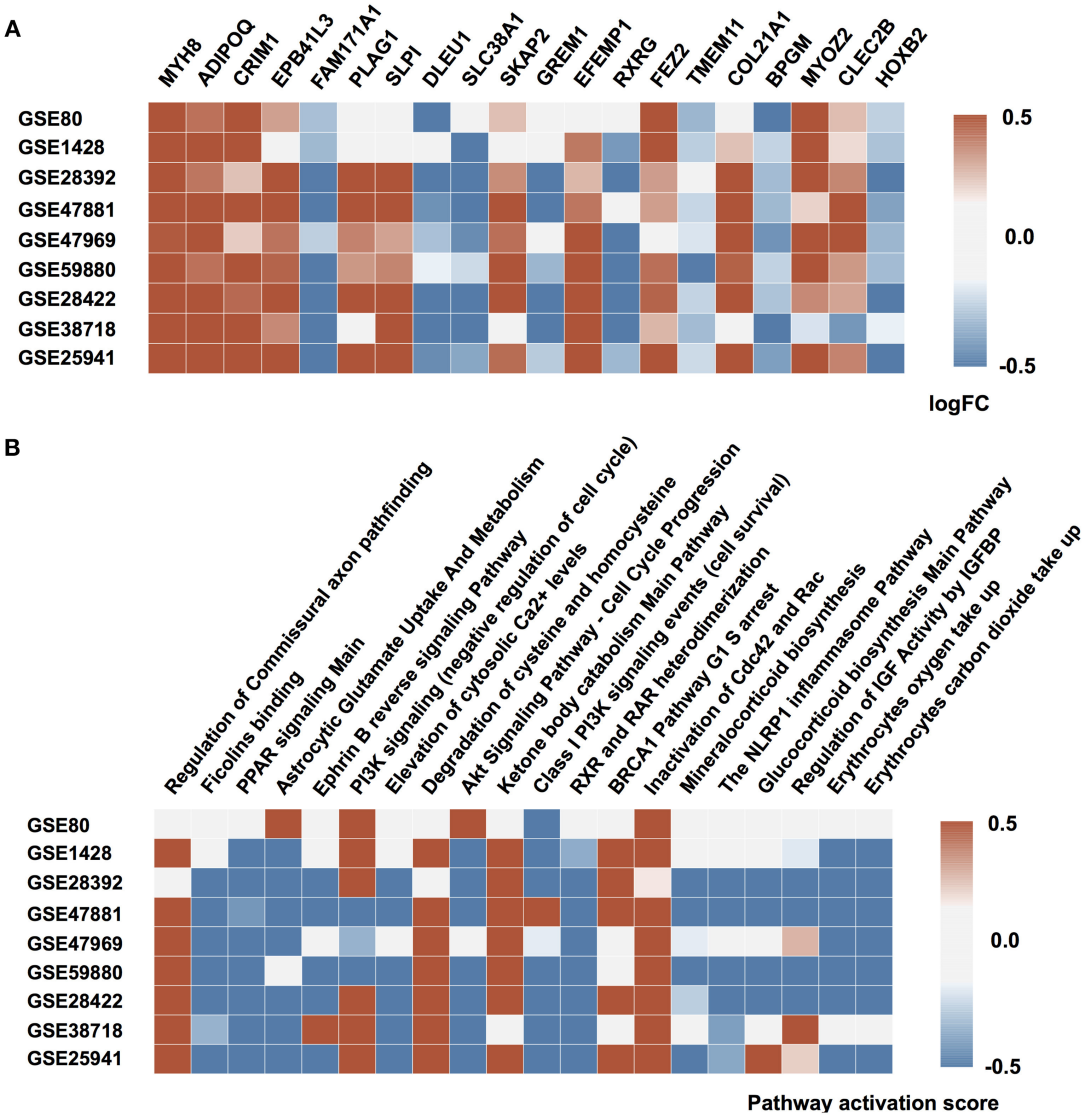
Molecular mechanisms of muscular aging. **(A)** Top 20 differentially expressed genes in “young” group against “old” group. **(B)** Signaling pathways perturbed in “young” group compared to “old” group. Up and down-regulated genes (pathways) are shown in red and blue respectively. The saturation of the color denotes to the perturbation amplitude.

It has been shown previously that muscle aging is strongly associated with compromised Ca^2+^ spark signaling and segregated intracellular Ca^2+^ release (Weisleder et al., 2006). Our data supports this observation. In particular, we observed a decreased expression of calcium ion binding protein EFEMP1 and sarcomeric protein MYOZ2 that binds to calcineurin, a phosphatase involved in calcium-dependent signal transduction, in the elderly group and corresponding activation of *Elevation of cytosolic* Ca^2+^ *levels Main Pathway*. Several other proteins directly or indirectly involved in sarcomere function and regulation are found in top20 perturbed gene list (Figure 2) including MYH8, EPB41L3 and SKAP2 (Pöllänen et al., 2010; Dreder et al., 2016). Interestingly, that decreased expression of tumor suppressor gene EPB41L3 that inhibits cell proliferation and promotes apoptosis was previously associated with cellular senescence in skin and lung (Yoon et al., 2004; Sembrat et al., 2016).

Another notable mechanism underlying aging-associated changes in muscle function is the irreversible change in fiber innervation (Holloszy and Carlson, 1995; Luff, 1998; Edström et al., 2007). Both FEZ2 necessary for normal axonal bundling and elongation within axon bundles and glutamine transporter SLC38A1 necessary for glutamate neurotransmitter cycling are down-regulated in aged muscle along with up-regulation of *Astrocytic Glutamate uptake* and down-regulation of *axon development* on the pathway level. While the decrease in oxygen saturation and glucose uptake also a play significant role in muscle aging, elevated expression of BPGM gene may mediate this effect. Moreover, dysregulation in BPGM expression is thought to play the similar role in age-related dementia (Kaminsky et al., 2013). Besides, the reduction in oxygen uptake is closely-related to overall mitochondrial function decline and increase in expression of TMEM11 gene responsible for mitochondrial morphogenesis (Short et al., 2005). The significant perturbation of *PPAR signaling* in the majority of data sets is also connected to impairment in glucose uptake and lipid metabolism during aging.

Surprisingly, pro-survival branches of the metabolic master-regulator signaling networks including *IGFR signaling* and *PI3K-Akt-mTOR axis* were down-regulated in young muscle comparing to the old ones. At the same time, the pathways associated with G1/S checkpoint arrest (*BRCA1 G1/S checkpoint arrest*) and ensuring long-lasting G0 state of the muscle cells were elevated in the samples from young donors. Several developmental genes (CRIM1, PLAG1, GREM1, and HOXB2) are found on top of the differentially expressed gene list. This observation may point to the age-associated tissue transition, e.g., muscular fibrosis.

An important cluster of aging-associated changes in muscular tissue refers to inflammation (Zoico et al., 2013). Specifically, CLEC2B gene, member of CTL/CTLD superfamily and one of the key inflammation and immune response regulators, is significantly perturbed in the majority of the datasets along with several inflammation-related pathways. Besides, the expression of SLPI gene responsible for resistance to viral, bacterial and fungal infections is down-regulated in the muscle samples of elderly individuals. Inflammation itself is closely tied up with detrimental changes in the extracellular matrix that contribute to muscle function decline (Kragstrup et al., 2011). Specific genes involved in extracellular matrix maintenance and experiencing the highest changes in expression profile include ADIPOQ and COL21A1.

Interestingly, that several genes that were not yet extensively studied in the context of muscle aging such as retinoid receptor RXRG, non-protein coding DLEU1 and very poorly described FAM171A1 are encountered in top20. We believe that these genes and their products may potentially represent novel biomarkers or therapeutic targets for age-related conditions in muscle.

### Age prediction

**Figure 3:**
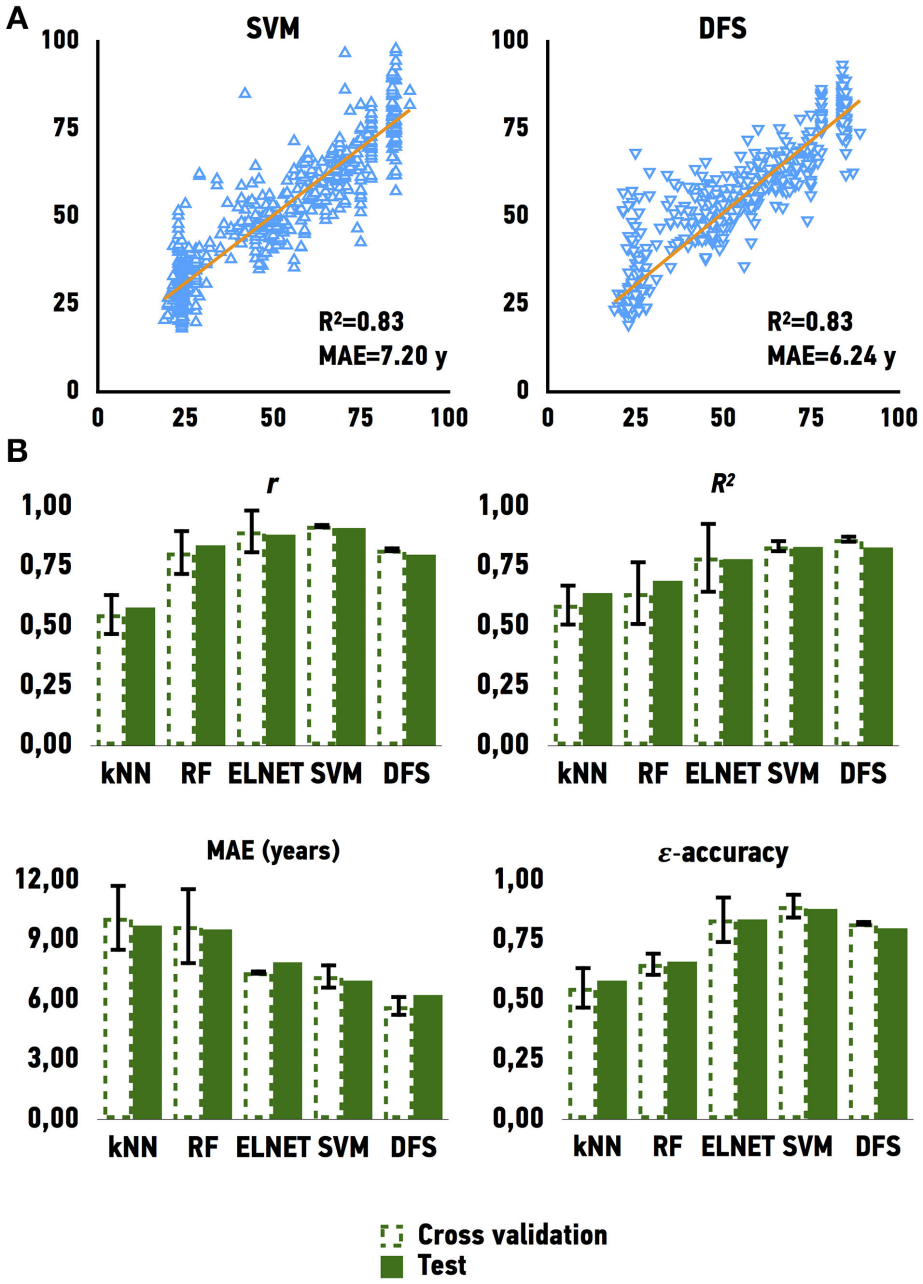
Performance of age predicting models **(A)** Actual chronological age vs. predicted age for Support Vector Machines model (SVM) and Deep Feature Selection Model (DFS) on validation and testing sets **(B)** Performance of models on validation and testing sets. *r* for Pearson correlation coefficient; *R*^2^ for coefficient of determination; MAE for mean absolute error, that shows the average disagreement between actual chronological and predicted ages; ε-accuracy the accuracy of prediction within a period, which was calculated for ε of 10 years, kNN, K Nearest Neighbors; RF, Random Forest; ELNET, ElasticNet; SVM, Support Vector Machines; DFS, Deep Feature Selection Models.

To develop an age predictor of samples we first explored a set of regression models. We used linear regression as a baseline model, which was compared to other machine learning methods such as Elastic Net, Support Vector Machines, k-Nearest Neighbors, Random Forest, and Deep Feature Selection Model. All models achieved a strong correlation of predicted and chronological age; however, both Support Vector Machines with a linear kernel and Deep Feature Selection model outperformed the other methods in age prediction, achieving *R*^2^ values of 0.83 and 0.83 and MAE values of 7.20 and 6.24 years, respectively (Figure 3 and Table 1). In comparison, the ElasticNet and Random Forest models achieved *R*^2^ values of 0.78 and 0.69, and MAE values of 7.37 and 9.54 years respectively. Lastly, the K-Nearest Neighbors model demonstrated an *R*^2^ of 0.64 and MAE of 9.73 years. Interestingly, the age of female samples tends to be predicted more accurately compared to male samples by all age predicting models (Table 1).

### External validation

**Figure 4:**
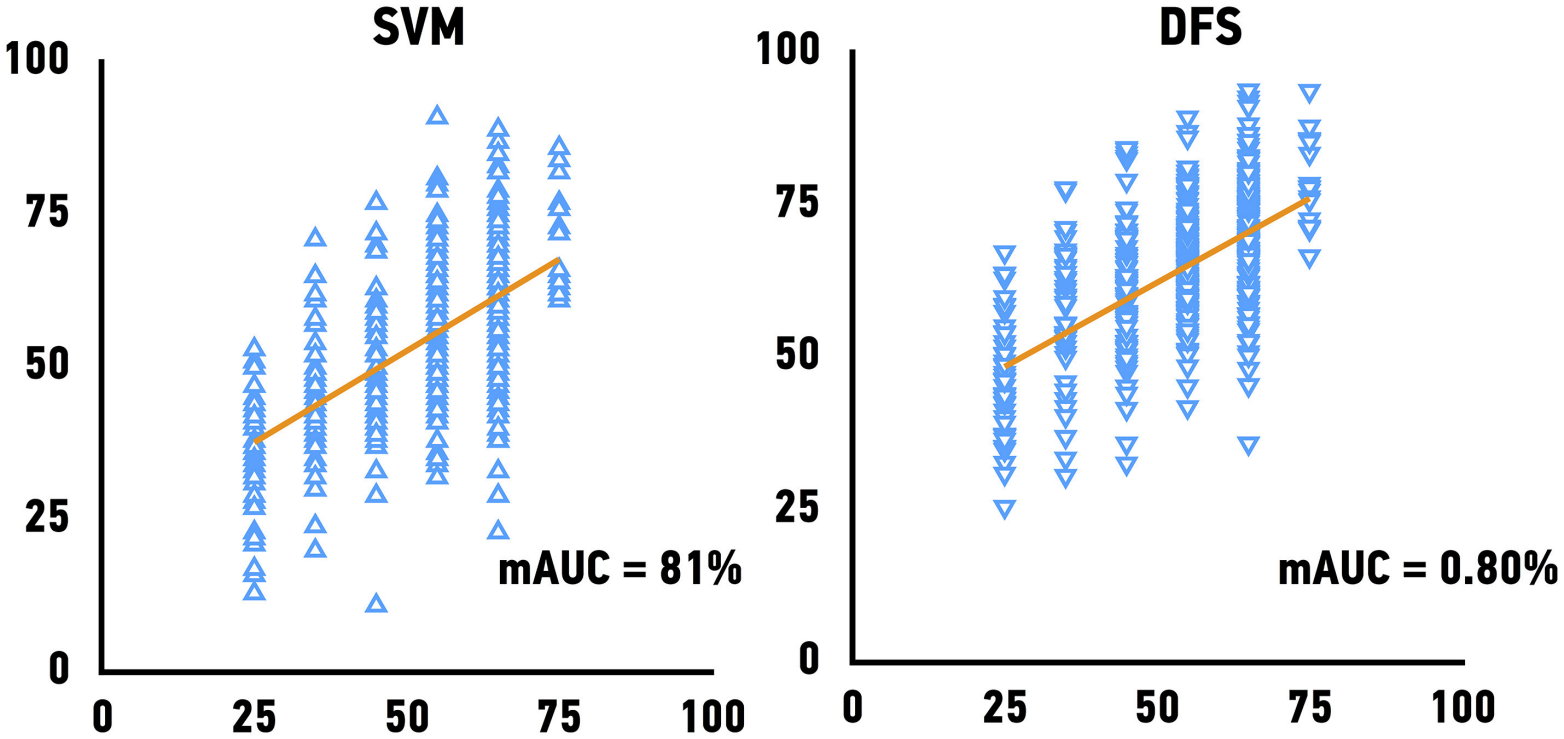
Performance of age predicting models on the external validation set. Mean of the actual chronological age bin vs. predicted age for Support Vector Machines models (SVM) and Deep Feature Selection Model (DFS).

The Genotype-Tissue Expression (GTEx) project dataset was used to validate our models. We predicted the age of skeletal muscle samples based on their gene expression profiles. Because GTEx project portal openly provide only age bin of donors, we have calculated mAUC (see Materials and Methods for details) to evaluate the accuracy of age group prediction. The previously best performing models, Support Vector Machines achieved mAUC of 0.80, compared to the mAUC of 0.90 on the original test set and Deep Feature Selection achieved mAUC of 0.80 and of respectively (Figure 4). The accuracy of age group prediction for male and female samples coincides with the performance on the test set and male samples tend to be predicted more accurately compare to female samples.

### Target identification

**Figure 5:**
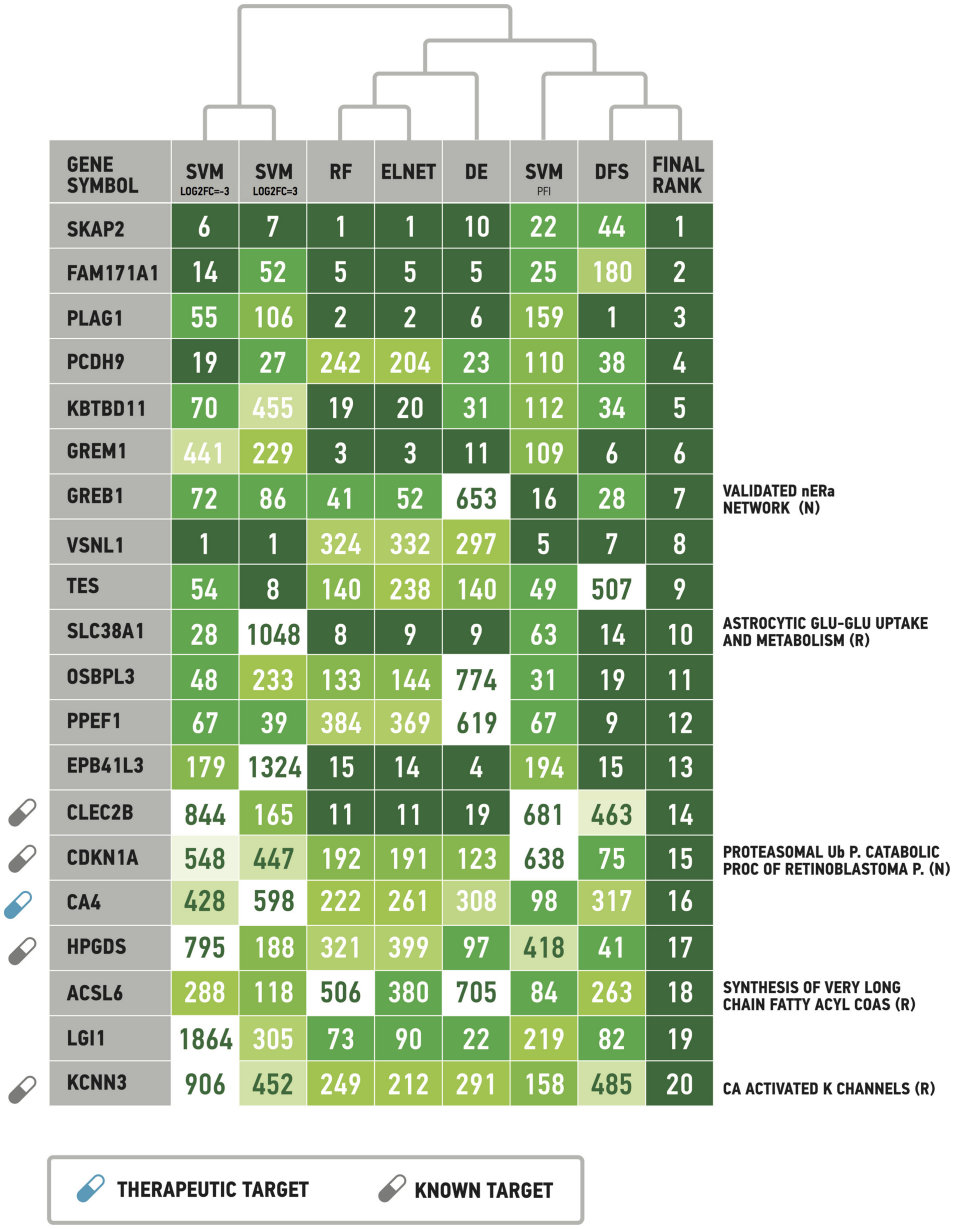
List of the most important genes selected by the Borda count algorithm applied over ranks assigned by Random Forest, ElasticNet, wrapper method applied over randomly permuted vectors of gene expression values (SVM_PFI_), increased values (SVM_log2FC = 3_) and decreased values (SVM_log2FC = −3_), Deep Feature Selection model (DFS) and the differential gene expression analysis (DE). See Materials and Method for details. Full signaling pathway names are supplied in the Table 2. Drug target information was obtained from DrugBank (www.drugbank.com). R, Reactome pathway database. N, NCI pathway database.

Following results on age prediction, we applied several feature importance analysis procedures to identify the genes most important for age prediction (see Materials and Methods for details). As different ranking methods return different values of relative importance, we used Borda count algorithm to summarize ranks and obtain final importance values (Table 2, Figure 5). Despite the fact that ranks of the selected top 20 genes vary, they all belong to the top 25% ranks of all genes. Interestingly, Random Forest and Elastic Net assigned similar ranks to the same genes. The wrapper method (applied over random permutations) and the Deep Feature Selection model demonstrate the closest results to the final ranking (Figure 5). At the same time, the wrapper method used over increased and decreased values showed different importance values and rank for the same genes, suggesting that the direction of changes in expression is important in age prediction for most of the genes analyzed. However, a number of genes including Src kinase associated phosphoprotein 2 (SKAP2), Visin like 1 (VSNL1) and Growth regulation by estrogen in breast cancer 1 (GREB1) demonstrated similar ranks in the context of both up-regulation and down-regulation.

**Table 2:** List of the most important genes selected by the Borda count algorithm applied over ranks assigned by Random Forest, ElasticNet, wrapper method applied over randomly permuted vectors of gene expression values (SVM_PFI_), increased values (SVM_log2FC = 3_) and decreased values (SVM_log2FC = −3_), Deep Feature Selection model (DFS) and the differential gene expression analysis (DE).

**Gene symbol**	**RF**	**ELNET**	**SVM _PFI_**	**SVM _log2FC = 3_**	**SVM _log2FC = –3_**	**DFS**	**DE**	**Final rank**	**Pathway**
SKAP2	1	1	22	6	7	44	10	1	
FAM171A1	5	5	25	14	52	180	5	2	
PLAG1	2	2	159	55	106	1	6	3	
PCDH9	242	204	110	19	27	38	23	4	
KBTBD11	19	20	112	70	455	34	31	5	
GREM1	3	3	109	441	229	6	11	6	
GREB1	41	52	16	72	86	28	653	7	Validated nuclear estrogen receptor alpha network Main Pathway (nci)
VSNL1	324	332	5	1	1	7	297	8	
TES	140	238	49	54	8	507	140	9	
SLC38A1	8	9	63	28	1048	14	9	10	Astrocytic glutamate glutamine uptake and metabolism main pathway (reactome)
OSBPL3	133	144	31	48	233	19	774	11	
PPEF1	384	369	67	67	39	9	619	12	
EPB41L3	15	14	194	179	1324	15	4	13	
CLEC2B	11	11	681	844	165	463	19	14	
CDKN1A	192	191	638	548	447	75	123	15	Regulation of retinoblastoma protein Pathway (proteasomal ubiquitin dependent protein catabolic process) (nci); Regulation of nuclear SMAD2 3 signaling Main Pathway; Regulation of retinoblastoman protein Main Pathway(nci)
CA4	222	261	98	428	598	317	308	16	
HPGDS	321	399	418	795	188	41	97	17	
ACSL6	506	380	84	288	118	263	705	18	Synthesis of very long chain fatty acyl CoAs Main Pathway (reactome)
LGI1	73	90	219	1864	305	82	22	19	
KCNN3	249	212	158	906	452	485	291	20	Ca activated K channels Main Pathway (reactome)

*See Materials and Method for details*.

While 5 out of the top 20 genes are known drug targets, some of the selected genes are known therapeutic targets, including the Carbonic anhydrase 4 (CA4) a target of anticovosculant drug, Topiramate, and a group of diuretics such as Chlorothiazide and Methazolamide. Recently, it has been shown that inhibition of CA4 effects relaxation of skeletal muscles both in model organisms (Wetzel et al., 2002; Tricarico et al., 2004) and human cells (Eguchi et al., 2006), suggesting their importance as potential drug targets in neuromuscular diseases.

## Discussion

This report described, to our knowledge, the first exhaustive signaling pathway analysis of skeletal human muscle that provides molecular insight into the differences among aged and young samples. Previously, transcriptomic analyses of muscle aging were conducted using the standard approach of gene expression analysis (Zahn et al., 2006; Sifakis et al., 2013). This study provides the first detailed pathway analysis involving the massive comparison of publicly available datasets consisting of both young and old muscle tissue. It also highlights the utility of pathway-based algorithms for dimension-reduction of high-dimensional transcriptomic data and for producing robust signatures of signaling pathway activation when comparing multiple cell states and types simultaneously.

Notably, the lists of important genes obtained using traditional differential expression analysis and machine learning methods while holding significant intersection, contain distinct genes that are both relevant for the condition under study. This emphasizes the potential benefits researchers could gain while using the proposed combined approach.

Hormonal imbalance and mitochondrial dysfunction are among the leading hallmarks of muscle aging identified by this study. On the signaling pathway level, elevation of cytosolic Ca^2+^, PPAR signaling and neurotransmitter recycling along with IGFR pathway activation accompanied by PI3K-Akt-mTOR signaling axis activation seen in the present analysis is believed to be key players in muscle growth, and as such dysregulation of these pathways very likely leads to a resulting decrease in muscle mass and regeneration ability (Yoon, 2017). Additionally, the impaired protein degradation demonstrated in the present analysis is also considered to be one of the key molecular mechanisms underlying sarcopenia (Lenk et al., 2010).

The best performing model used in the present analysis, a feed-forward neural network, achieved an MAE of 6.24 years, demonstrating reasonably good accuracy in terms of age prediction. Notably, female samples tend to be predicted more accurately, which is in line with our previous findings in age predction by blood biochemistry (Mamoshina et al., 2018). Indeed previous analysis highlighted sex-specificity of muscle aging transcriptional profiles (Liu et al., 2013) and at the same time model organisms and human studies also demonstrated the sex-dependent differences in aging rates (Waisman et al., 2013; Horvath et al., 2016).

Previously, Sood et al. applied supervised machine learning algorithm (K-Nearest Neighbors) in order to perform binary classification muscle gene expression profiles by “young” and “old” achieving an average AUC of 93% (70–100%) for independent muscle data (Sood, 2015). Here we present more complex approach, allowing to quantify aging changes. Our current results show that the best performing model could achieve 0.80 mAUC (for 6 age bin groups) on the massive external validation set provided by the GTEx project (*n* = 564).

Furthermore, our results show that age prediction models can be used as a tool for identifying perspective targets for anti-aging therapies, and can serve as a potential panel of companion biomarkers for evaluating the effect of such therapies. Using transcriptional signatures, the general approach encapsulated by the present study could be further applied to other tissues and other disease areas.

## Author contributions

AZ, IO, and PM planned the study. MV and ES conducted original data screening and preparation. IO conducted and interpreted gene and pathway analysis. PM, MV, and EL conducted and interpreted machine learning analysis. FC helped review the manuscript and references. PM, MV, IO, and AZ conducted further data evaluation and manuscript preparation.

### Conflict of interest statement

PM, MV, IO, EL, ES, and AZ are associated with the company, Insilico Medicine, Inc., engaged in drug discovery and aging research. The remaining author declares that the research was conducted in the absence of any commercial or financial relationships that could be construed as a potential conflict of interest.

## Supplementary material

Table S1 A list of datasets analyzed in this study.

Table S2 A list of genes analyzed in this study.
